# Gait Characteristics and Fall Risk in Amyotrophic Lateral Sclerosis: A 12-Month Prospective Cohort Study

**DOI:** 10.1159/ndd/aduag003

**Published:** 2026-09-22

**Authors:** Nan Hu, Ning Su, Dingding Zhang, Jiangxia Zhang, Kai Wang, Yuming Xu, Zhenzhong Li, Lihua Wang, Bo Wu, Lan Chu, Yinzhou Wang, Hong Jiang, Zhengqi Lu, Jian Wu, Xiangmin Fan, Fei Han, Jing Yuan, Yicheng Zhu, Mingsheng Liu

**Affiliations:** a Department of Neurology, Peking Union Medical College Hospital, Chinese Academy of Medical Sciences, Beijing, China; b School of Biomedical Engineering, Capital Medical University, Beijing, China; c Department of Neurology, The First Affiliated Hospital of Zhengzhou University, Zhengzhou, China; d Department of Neurology, The Second Hospital of Hebei Medical University, Shijiazhuang, China; e Department of Neurology, Beijing Tiantan Hospital, Capital Medical University, Beijing, China; f Department of Neurology, West China Hospital, Sichuan University, Chengdu, China; g Department of Neurology, The First Affiliated Hospital of Guangxi Medical University, Nanning, China; h Department of Neurology, The First Affiliated Hospital of Fujian Medical University, Fuzhou, China; i Department of Neurology, Xiangya Hospital, Central South University, Changsha, China; j Department of Neurology, The First Affiliated Hospital of Sun Yat-sen University, Guangzhou, China; k Department of Neurology, Huashan Hospital, Fudan University, Shanghai, China; l Beijing CAS-Ruiyi Information Technology Co., Ltd., Beijing, China

**Keywords:** Amyotrophic lateral sclerosis, Gait analysis, Falls, Timed Up and Go test, Predictive model, Wheelchair use

## Abstract

**Introduction:**

Gait impairment in amyotrophic lateral sclerosis (ALS) substantially increases fall risk and accelerates functional decline. However, prospective data linking quantitative gait parameters to fall outcomes remain scarce. We aimed to identify gait parameters associated with falls and functional decline in ALS.

**Methods:**

In this single-center prospective cohort study, we enrolled 256 ALS patients and 256 matched healthy controls. Nineteen spatiotemporal gait parameters were captured using a 3D vision-based system at baseline. Falls, time to wheelchair use, and ALSFRS-R decline were recorded over 12 months. Multivariable logistic regression was used to identify independent fall predictors.

**Results:**

During follow-up, 129 of 224 patients (57.6%) experienced at least one fall. Prolonged Timed Up and Go (TUG) time (OR = 1.083 per second, 95% CI, 1.036–1.133, *p* < 0.001), younger age (OR = 0.968 per year, 95% CI, 0.940–0.996, *p* = 0.024), and lower MRC sum score (OR = 0.976 per point, 95% CI, 0.954–0.998, *p* = 0.035) independently predicted falls. The model showed moderate discrimination (AUC = 0.665, 95% CI, 0.594–0.736). Gait speed (*r*_s_ = 0.366) and TUG time (*r*_s_ = −0.381) correlated most strongly with time to wheelchair use (both *p* < 0.001), whereas no gait parameters correlated with ALSFRS-R decline rate.

**Conclusion:**

TUG time, younger age, and lower MRC score independently predict falls in ALS, though with modest predictive accuracy. The modest model performance underscores the multifactorial nature of falls and the need for multi-domain risk assessment. Gait speed and TUG time may warrant further investigation as candidate surrogate markers for functional decline. Future studies integrating cognitive evaluation, continuous monitoring, and attention to psychosocial factors may improve prediction and fall prevention strategies.

## Introduction

Amyotrophic lateral sclerosis (ALS) is a progressive neurodegenerative disorder affecting both upper and lower motor neurons, leading to muscle weakness, spasticity, and functional disability [1]. Gait impairment is a hallmark of ALS, significantly impacting quality of life and increasing fall risk [2]. Falls in ALS can result in fractures, head trauma, and accelerated functional decline, contributing to morbidity and mortality [3, 4].

Previous studies have documented gait alterations in ALS, including reduced gait speed, shortened stride length, widened step width, and prolonged double support time [5, 6]. However, most studies have been cross-sectional, and the prospective relationship between quantitative gait parameters and fall risk remains poorly characterized. The revised ALS Functional Rating Scale (ALSFRS-R) provides a global measure of functional status but lacks the granularity to capture subtle gait abnormalities or predict falls [7].

Recent advances in three-dimensional motion capture and deep learning have enabled automated, high-precision gait analysis [8, 9]. Our team previously developed a deep learning-based gait analysis system using the Azure Kinect camera and established a multi-center normative database. In a prior cross-sectional study, we identified distinct gait patterns associated with upper motor neuron (UMN) involvement in ALS [10]. However, whether these baseline gait features predict future falls or functional decline requires prospective validation.

Cognitive and behavioral impairments are increasingly recognized as integral features of ALS that may influence gait and fall risk. Executive dysfunction, in particular, has been associated with increased gait variability and higher fall rates in ALS and other neurodegenerative conditions [11]. However, routine cognitive screening is not universally incorporated into ALS clinical assessments, and the independent contribution of cognitive status to fall prediction remains incompletely characterized.

In this prospective cohort study, we aimed to (1) characterize gait abnormalities in ALS using quantitative analysis; (2) identify baseline gait parameters associated with incident falls during 12-month follow-up; (3) develop and evaluate a predictive model for fall risk; and (4) explore associations between gait parameters and time to wheelchair use.

## Methods

### Study Design

This was a single-center, prospective, observational cohort study.

### Participants

#### ALS Patients

Consecutive patients diagnosed with definite or probable ALS according to the revised El Escorial criteria [12] (including probable and probable laboratory-supported categories) were recruited from the ALS clinic at Peking Union Medical College Hospital between April 2024 and October 2025. The revised El Escorial criteria were used as these remain the most widely adopted framework internationally and ensure consistency with our previous work [10]. Inclusion criteria were (1) age ≥18 years; (2) ability to complete a 3-meter walk test; and (3) forced vital capacity ≥50% predicted at baseline, to ensure sufficient respiratory reserve to tolerate the physical demands of the gait test battery, consistent with our previous work [10]. Exclusion criteria included (1) other neurological or orthopedic conditions affecting gait; (2) significant cognitive impairment preventing understanding of test instructions.

All patients were managed through a specialized multidisciplinary ALS clinic at Peking Union Medical College Hospital, which provides standardized care including regular neurological assessments, respiratory function monitoring, nutritional support, and rehabilitation consultations. Physiotherapy and fall prevention education were offered as part of routine clinical care, but specific interventions were not standardized as part of this observational study and were not systematically recorded.

All patients underwent comprehensive clinical assessment including demographics, disease characteristics (onset site, duration), ALSFRS-R [7], Medical Research Council (MRC) sum score (12 muscle groups including bilateral shoulder abduction, elbow flexion, elbow extension, wrist flexion, wrist extension, thumb abduction, little finger abduction, hip flexion, knee flexion, knee extension, ankle dorsal extension, ankle plantar flexion, total score 0–120), and Penn UMN score (range 0–18) [13]. We used a modified Penn UMN score (range 0–18) assessing global UMN burden, including tendon reflexes, pathological reflexes, and muscle tone, as previously described [10, 13].

#### Healthy Controls

Healthy control data were obtained from an unpublished nationwide multi-center database (September 2021-March 2023) comprising 1,796 individuals with no self-reported neurological, musculoskeletal, or gait-affecting conditions. Propensity score matching (1:1) based on age, sex, and BMI was performed to derive a matched control group of 256 participants for case-control comparisons. Detailed methods for control recruitment and matching have been described previously [10].

### Gait Acquisition and Processing

Gait assessment was performed using the Ready-GO system (Beijing CAS-Ruiyi Information Technology Co., Ltd.), a medical device certified by China's National Medical Products Administration. The system employs a Microsoft Azure Kinect depth camera (30 Hz sampling frequency) to capture whole-body movement, reconstructing a 3D skeletal model (25 joints) in real-time. A deep learning-based pipeline (bidirectional long short-term memory networks) automatically identifies key gait events (heel strike, toe-off) and calculates gait parameters with high accuracy. The detailed methodology for gait acquisition, testing protocol, and parameter definitions has been published previously [10].

### Testing Protocol

All gait assessments were performed by trained research staff (a neurologist and a physiotherapist, each with >3 years of experience in neurodegenerative disease gait analysis). Operators completed a standardized training protocol on the Ready-GO system, including system operation, patient positioning, and quality control procedures, before conducting independent assessments. Inter-rater reliability for key gait parameters was previously established (ICC range 0.78–0.92) [10].

Participants completed a standardized test battery in a well-lit room: (1) 3-meter walk test (repeated three times, with steady-state strides averaged); (2) Timed Up and Go (TUG) test; (3) five-times sit-to-stand (FTSTS) test; (4) straight-line walking (10 steps); and (5) hand pro-nation-supination test (left/right). At least 1 min of rest was provided between trials.

### Gait Parameters

Nineteen spatiotemporal gait parameters were derived and grouped into five functional domains: (1) basic spatiotemporal: gait speed, stride length (left/right), stride speed (left/right), cadence (left/right), step height (left/right), step width; (2) postural stability: stance phase (left/right), double support phase, turn time, TUG time, stance phase asymmetry; (3) rhythm and coordination: swing phase (left/right), swing speed (left/right), stride asymmetry index, gait cycle time asymmetry index; (4) functional tasks: FTSTS time, FTSTS sway, hand pro-nation-supination time (left/right), straight-line walking time. Definitions of all parameters are provided in online supplemental Table 1 (for all online suppl. material, see https://doi.org/10.1159/ndd/aduag003) and our previous publication [10].

### Follow-Up Protocol

Follow-up was initiated from the baseline enrollment visit, which coincided with the comprehensive gait assessment at our center. For most patients, this occurred at their first visit to our ALS clinic; for patients diagnosed elsewhere, this represented the time of their first comprehensive evaluation at our center. Patients were followed prospectively for 12 months. Follow-up visits were scheduled at 6 and 12 month post-enrollment. For patients unable to attend in-person visits, structured telephone interviews or online consultations were conducted. The following information was collected: (1) number of falls (defined as any unintentional change in balance resulting in contact with the ground, wall, furniture, or other surface [4]); (2) time to full-time wheelchair use; (3) invasive mechanical ventilation or death; (4) ALSFRS-R score; (5) MRC sum score (for in-person visits). The endpoint was defined as completion of 12-month follow-up, death, or initiation of invasive mechanical ventilation.

### Outcomes

Primary outcome: occurrence of falls (binary: ≥1 fall vs. no falls) and total fall count during 12-month follow-up. Secondary outcomes: (1) time from baseline to full-time wheelchair use (months), defined as the point at which the patient began using a wheelchair for all ambulation outside of bed, including within the home. Patients who used a wheelchair only for long distances but continued to walk short distances were not classified as having reached this endpoint; (2) ALSFRS-R decline rate: (baseline score-follow-up score)/follow-up duration (months). For patients who reached endpoints, the last observation carried forward (LOCF) method was applied.

### Statistical Analysis

#### Sample Size

Based on our center’s historical annual caseload (approximately 150–180 new ALS diagnoses per year), we planned to consecutively enroll approximately 250 patients during the 18-month recruitment period. A total of 256 patients were finally included.

#### Software and Significance Level

Analyses were performed using IBM SPSS Statistics (version 26.0) and JASP (version 0.19.2). Significance was set at two-tailed *p* < 0.05. For multiple comparisons, the Benjamini-Hochberg false discovery rate correction was applied, with q < 0.05 considered significant.

#### Normality Testing

Continuous variables were tested for normality using the Shapiro-Wilk test. Normally distributed variables are presented as mean ± standard deviation (SD) and compared using independent samples *t*-tests or ANOVA; non-normally distributed variables are presented as median (interquartile range [IQR]) and compared using Mann-Whitney U or Kruskal-Wallis *H* tests. Categorical variables are presented as frequencies (%) and compared using chi-square or Fisher's exact tests.

#### Comparison between ALS and HC

As the methods for case-control comparisons, stratified analyses, and correlation analyses largely overlap with our previous publication [10], these are briefly summarized here. Detailed results for ALS vs. HC comparisons and stratified analyses are provided in online supplementary Tables 3–7.

#### Comparison between Fallers and Non-Fallers

Patients who experienced at least one fall during follow-up were classified as fallers. Baseline gait parameters were compared between fallers and non-fallers using Mann-Whitney U tests with false discovery rate correction.

#### Correlation Analysis

Spearman rank correlation coefficients were calculated between baseline gait parameters and (1) total fall count, (2) time to wheelchair use, and (3) ALSFRS-R decline rate. Correlation strength was defined as follows: |*r*_s_|<0.3 weak, 0.3≤|*r*_s_|<0.6 moderate, |*r*_s_|≥0.6 strong.

#### Multivariable Logistic Regression for Fall Prediction

A binary logistic regression model (forward stepwise, likelihood ratio) was constructed to identify independent predictors of falls. Candidate variables included all gait parameters significantly associated with falls in univariate analysis, along with age, sex, MRC sum score, ALSFRS-R, and Penn UMN score. Model calibration was assessed using the Hosmer-Lemeshow test, and discrimination was evaluated using the area under the receiver operating characteristic curve (AUC). We acknowledge that forward stepwise selection has limitations, including potential instability in variable selection and a tendency to overfit. However, we chose this approach for its simplicity and interpretability in a clinical context, and we complemented it with sensitivity analyses to assess model stability. Penalized regression methods (e.g., LASSO) were not employed due to the limited event count and the exploratory nature of this analysis. We recognize this as a methodological limitation and discuss it in Limitations.

#### Sensitivity and Subgroup Analyses

Sensitivity analyses were performed by (1) redefining falls as “≥2 falls;” (2) excluding patients using walking aids at baseline; (3) imputing missing data using LOCF. Subgroup analyses were stratified by Penn UMN score, onset site, disease duration, and baseline gait speed. We further explored the interaction between age and UMN burden by stratifying patients by both age (<57 vs. ≥57 years) and Penn UMN score (<6 vs. ≥6).

## Results

### Participant Characteristics

A total of 256 ALS patients (135 males, 121 females) with mean age 57.37 ± 10.46 years and median disease duration 9 (7–15) months were enrolled. Bulbar-onset cases accounted for 63 (24.6%). Propensity score matching yielded 256 well-balanced healthy controls (age *p* = 0.896, sex *p* = 0.989, BMI *p* = 0.365). Baseline characteristics are shown in Table 1. The median ALSFRS-R respiratory subscore (items 10–12) was 10 (IQR 10–12) out of a maximum of 12, indicating well-preserved respiratory function at baseline, consistent with our inclusion criterion of FVC ≥50% predicted.

**Table 1.** Baseline demographic and clinical characteristics of ALS patients and matched healthy controls

Variable	ALS (*n* = 256)	HC (*n* = 256)^a^	*p* value
Age, years	57.37±10.46	57.25±10.41	0.896
Gender, M/F	135/121	136/120	0.989
BMI, kg/m^2^	23.70±3.74	23.44±2.89	0.365
Disease duration, months	9 (7–15)	​	​
Bulbar onset, *n* (%)	63 (24.61%)	​	​
ALSFRS-R score	40 (37–44)	​	​
ALSFRS-R respiratory subscore	10 (10–12)	​	​
MRC sum score	101 (90–111)	​	​
Penn UMN score^b^	6 (3–10)	​	​
Lower limb involvement^c^, *n* (%)	167 (65.23%)	​	​

^a^ HC matched by propensity score (1:1) from a nationwide multi-center database (*N* = 1,792).

^b^ Penn UMN score: modified version (range 0–18), assessing global UMN burden including tendon reflexes, pathological reflexes, and muscle tone, as previously described [10].

^c^ Lower limb involvement defined as lower limb MRC subscore <40 (out of 50).

Normally distributed variables are shown as mean ± SD; non-normally distributed variables as median (IQR). Group comparisons: independent *t*-test for age and BMI; chi-square for gender. All comparisons showed no significant differences (*p* > 0.05).

During follow-up, 32 patients (12.5%) were lost to follow-up. Of the 224 patients who completed the full 12-month follow-up or reached a study endpoint, 172 survived, 31 died, and 21 required invasive ventilation. Since only 52 patients (20.3%) reached the composite endpoint of death or ventilation within 12 months, the median survival time was not estimable. The median ALSFRS-R decline rate during follow-up was 0.75 (IQR 0.33–2.08) points per month. Using ΔALSFRS-R <1.00 points per month as the definition of slow progression, 126 patients (56.25%) were classified as slow progressors.

Comparison of baseline characteristics between the 224 patients who completed follow-up and the 32 patients lost to follow-up revealed no significant differences in age, sex, ALSFRS-R score, or TUG time (all *p* > 0.05), suggesting that attrition did not introduce substantial selection bias.

### Gait Abnormalities in ALS Compared to Healthy Controls

Consistent with our prior cross-sectional findings [10], ALS patients demonstrated significant gait abnormalities across all functional domains compared to HC, including reduced gait speed, shortened stride length, decreased cadence, widened step width, prolonged double support and TUG times, and reduced swing speed (all *q* < 0.001) (Online suppl. Table 2). Effect sizes (r) were largest for cadence (left: −0.718, right: −0.716), stride speed (left: −0.581, right: −0.586), and swing speed (left: −0.514, right: −0.512). Stratified analyses by age, sex, MRC score, ALSFRS-R, and Penn UMN score are presented in online supplementary Tables 3–7.

### Comparison of Gait Parameters between Fallers and Non-Fallers

Among the 224 patients who completed follow-up, 129 (57.6%) experienced at least one fall during the 12-month period. Baseline gait parameters were compared between fallers and non-fallers (Table 2). Fallers exhibited slower gait speed, shorter stride length, lower stride speed, lower cadence, lower step height, prolonged TUG time, prolonged double support phase, greater gait cycle time asymmetry, shorter swing phase, lower swing speed, and longer left hand pro-nation-supination time (all *q* < 0.05). Step width, turn time, and FTSTS time did not differ significantly between groups.

**Table 2.** Comparison of baseline gait parameters between fallers and non-fallers during 12-month follow-up

Gait parameter		Non-fallers (*n* = 95)	Fallers (*n* = 129)	*p* value	q (FDR)	Effect size (r)
Basic spatiotemporal	Gait speed (m/s)	0.9 (0.8,1.0)	0.8 (0.4,1.0)	**0.010**	**0.018**	0.172
	Left stride (m)	1.1 (1.0,1.2)	1.0 (0.7,1.2)	**0.008**	**0.016**	0.177
	Right stride (m)	1.1 (1.0,1.2)	1.0 (0.7,1.2)	**0.019**	**0.029**	0.157
	Left stride speed (m/s)	0.9 (0.8,1.0)	0.8 (0.5,1.0)	**0.008**	**0.016**	0.176
	Right stride speed (m/s)	0.9 (0.8,1.0)	0.8 (0.5,1.0)	**0.008**	**0.016**	0.176
	Left cadence (step/min)	100.0 (94.7,112.5)	100.0 (85.7,105.9)	**0.009**	**0.017**	0.175
	Right cadence (step/min)	100.0 (90.0.112.5)	94.7 (85.7,105.9)	**0.001**	**0.003**	0.223
	Left step height (m)	0.1 (0.1,0.1)	0.1 (0.1,0.1)	**0.012**	**0.020**	0.168
	Right step height (m)	0.1 (0.1,0.1)	0.1 (0.1,0.1)	**0.019**	**0.029**	0.157
	Step width (m)	0.1 (0.1,0.1)	0.1 (0.1,0.2)	0.118	0.159	−0.104
Postural stability	Left stance (%)	67.7 (66.7,69.4)	68.4 (66.9,72.2)	0.086	0.129	−0.115
	Right stance (%)	68.0 (66.7,69.4)	69.0 (67.4,73.1)	**0.007**	**0.015**	−0.180
	Double support (%)	36.2 (34.4,38.3)	37.3 (34.5,46.4)	**0.029**	**0.039**	−0.146
	Turn time (s)	1.4 (1.1,1.7)	1.4 (1.1,1.8)	0.358	0.429	−0.061
	TUG time (s)	12.4 (10.5,14.7)	15.7 (11.5,24.2)	**<0.001**	**<0.001**	−0.277
Rhythm and coordination	Stride asymmetry (%)	0.9 (0.4,1.8)	1.0 (0.5,2.0)	0.581	0.627	−0.037
	Gait cycle asymmetry (%)	5.9 (1.0,6.9)	6.4 (3.5,9.3)	**0.036**	**0.045**	−0.140
	Stance asymmetry (%)	1.1 (0.5,2.2)	1.5 (0.8,2.7)	0.081	0.126	−0.117
	Left swing (%)	32.3 (30.6,33.3)	31.6 (27.8,33.1)	**0.031**	**0.042**	0.144
	Right swing (%)	32.0 (30.6,33.3)	31.0 (26.9,32.6)	**0.003**	**0.007**	0.200
	Left swing speed (m/s)	2.3 (2.1,2.5)	2.1 (1.5,2.4)	**0.004**	**0.009**	0.194
	Right swing speed (m/s)	2.3 (2.1,2.5)	2.1 (1.4,2.5)	**0.016**	**0.025**	0.162
Functional tasks	FTSTS time (s)	12.1 (9.6,16.2)	11.9 (9.2,15.8)	0.533	0.599	0.042
	FTSTS sway (%)	32.9 (28.0,40.8)	34.2 (27.9,44.9)	0.539	0.599	−0.041
	Left hand pro-nation-supination (s)	10.2 (8.7,12.4)	10.9 (9.1,14.0)	**0.033**	**0.041**	−0.142
	Right hand pro-nation-supination (s)	10.0 (8.3,11.8)	10.1 (8.7,12.0)	0.239	0.307	−0.079
	Straight-line walking (s)	12.3 (9.7,14.8)	12.3 (9.9,16.5)	0.787	0.787	−0.018

Data presented as median (IQR). Group comparisons: Mann-Whitney U test. FDR-corrected *q* < 0.05 considered significant and values in bold. Effect size r = Z/√N (N = 224).

### Correlation of Gait Parameters with Fall Count, Time to Wheelchair Use, and ALSFRS-R Decline Rate

Spearman correlation analyses (Table 3) revealed that 17 gait parameters significantly correlated with fall count, all with weak effect sizes (|*r*_s_|<0.3). The strongest correlations with fall count were observed for TUG time (*r*_s_ = 0.275, *p* < 0.001) and right swing phase (*r*_s_ = −0.234, *p* < 0.001).

**Table 3.** Spearman correlations between baseline gait parameters and 12-month outcomes (fall count, time to wheelchair use, and ALSFRS-R decline rate)

Gait parameter		Fall count		Time to wheelchair use		ALSFRS-R decline rate	
		*r*_s_	*p* value	*r*_s_	*p* value	*r*_s_	*p* value
Basic spatiotemporal	Gait speed (m/s)	−0.199	**0.003**	0.366	**<0.001**	0.021	0.757
	Left stride (m)	−0.203	**0.002**	0.355	**<0.001**	0.065	0.338
	Right stride (m)	−0.184	**0.006**	0.355	**<0.001**	0.062	0.363
	Left stride speed (m/s)	−0.201	**0.003**	0.381	**<0.001**	0.019	0.780
	Right stride speed (m/s)	−0.198	**0.003**	0.376	**<0.001**	0.021	0.754
	Left cadence (step/min)	−0.147	**0.029**	0.327	**<0.001**	−0.050	0.463
	Right cadence (step/min)	−0.193	**0.004**	0.306	**<0.001**	−0.063	0.353
	Left step height (m)	−0.181	**0.007**	0.243	**<0.001**	−0.001	0.987
	Right step height (m)	−0.154	**0.022**	0.235	**<0.001**	−0.044	0.516
	Step width (m)	0.145	**0.032**	−0.169	**0.012**	−0.031	0.647
Postural stability	Left stance (%)	0.170	**0.012**	−0.354	**<0.001**	−0.022	0.751
	Right stance (%)	0.232	**<0.001**	−0.349	**<0.001**	0.019	0.779
	Double support (%)	0.184	**0.006**	−0.339	**<0.001**	−0.024	0.726
	Turn time (s)	0.124	0.065	−0.015	0.821	−0.051	0.448
	TUG time (s)	0.275	**<0.001**	−0.381	**<0.001**	−0.024	0.721
Rhythm and coordination	Stride asymmetry (%)	−0.024	0.726	0.016	0.812	−0.050	0.458
	Gait cycle asymmetry (%)	0.159	**0.018**	−0.154	**0.022**	0.039	0.568
	Stance asymmetry (%)	0.052	0.444	−0.220	**0.001**	0.042	0.533
	Left swing (%)	−0.173	**0.010**	0.359	**<0.001**	0.018	0.795
	Right swing (%)	−0.234	**<0.001**	0.351	**<0.001**	−0.023	0.731
	Left swing speed (m/s)	−0.219	**0.001**	0.384	**<0.001**	−0.004	0.949
	Right swing speed (m/s)	−0.187	**0.005**	0.362	**<0.001**	0.020	0.763
Functional tasks	FTSTS time (s)	−0.023	0.739	−0.066	0.329	0.035	0.605
	FTSTS sway (%)	0.114	0.091	−0.080	0.237	−0.064	0.344
	Left hand pro-nation-supination (s)	0.091	0.180	−0.089	0.188	0.124	0.066
	Right hand pro-nation-supination (s)	0.029	0.670	−0.049	0.474	0.107	0.114
	Straight-line walking (s)	0.005	0.938	−0.169	0.012*	0.059	0.385

Significant *p* values (*p* < 0.05) are indicated in bold. *r*_s_: Spearman rank correlation coefficient.

Notably, gait parameters demonstrated stronger correlations with time to wheelchair use (Table 3). Faster gait speed (*r*_s_ = 0.366, *p* < 0.001) and shorter TUG time (*r*_s_ = −0.381, *p* < 0.001) were most strongly associated with longer time to wheelchair use. Other significantly correlated parameters included stride length, stride speed, cadence, step height, stance phase, double support phase, swing phase, swing speed, and stance phase asymmetry (all *p* < 0.05), with correlation coefficients ranging from |*r*_s_| = 0.154–0.384.

No gait parameters significantly correlated with ALSFRS-R decline rate (all *p* > 0.05). We further examined correlations between gait parameters and individual ALSFRS-R gross motor subscore items (walking, climbing stairs, dressing, and hygiene). As expected, gait parameters showed the strongest correlations with the walking and stair-climbing items (*r*_s_ ranging from 0.18 to 0.41, all *p* < 0.01), while correlations with dressing and hygiene items were weak and non-significant (all *p* > 0.05). This pattern supports the specificity of quantitative gait parameters as markers of ambulatory function rather than global disability (online suppl. Table 8).

### Predictive Model for Falls

Multivariable logistic regression (forward stepwise, likelihood ratio) identified three independent predictors of falls: TUG time, age, and MRC sum score (Table 4). Collinearity diagnostics using variance inflation factors (VIFs) showed no evidence of multicollinearity among the final model variables (TUG time: VIF = 1.42; age: VIF = 1.18; MRC sum score: VIF = 1.33; all VIF <5). The model demonstrated good calibration (Hosmer-Lemeshow χ^2^ = 5.267, df = 8, *p* = 0.729) but modest discrimination (Nagelkerke *R*^2^ = 0.173). Overall classification accuracy was 65.9%, with sensitivity of 75.8% and specificity of 52.2%. ROC analysis yielded an AUC of 0.665 (95% CI, 0.594–0.736, *p* < 0.001) (Fig. 1). Prolonged TUG time (OR = 1.083 per second, 95% CI, 1.036–1.133, *p* < 0.001), lower MRC score (OR = 0.976 per point, 95% CI, 0.954–0.998, *p* = 0.035), and younger age (OR = 0.968 per year, 95% CI, 0.940–0.996, *p* = 0.024) independently increased fall risk.

**Table 4.** Multivariable logistic regression for predicting fall risk in ALS patients (forward stepwise, likelihood ratio)

Variable	B	SE	Wald χ^2^	*p* value	OR	95% CI
TUG time (s)	0.080	0.023	12.267	<0.001	1.083	1.036–1.133
Age (years)	−0.033	0.015	5.112	0.024	0.968	0.940–0.996
MRC sum score	−0.024	0.012	4.453	0.035	0.976	0.954–0.998
Constant	3.388	1.446	5.489	0.019	29.610	–

Model performance: Collinearity diagnostics: TUG time VIF=1.42; age VIF=1.18; MRC sum score VIF=1.33 (all VIF <5). Hosmer-Lemeshow χ^2^ = 5.267, df = 8, *p* = 0.729; Nagelkerke *R*^2^ = 0.173; overall classification accuracy = 65.9%; sensitivity = 75.8%; specificity = 52.2%; AUC=0.665 (95% CI, 0.594–0.736). Candidate variables included all gait parameters significantly associated with falls in univariate analysis, along with age, sex, MRC score, ALSFRS-R, and Penn UMN score.

**Fig. 1.**
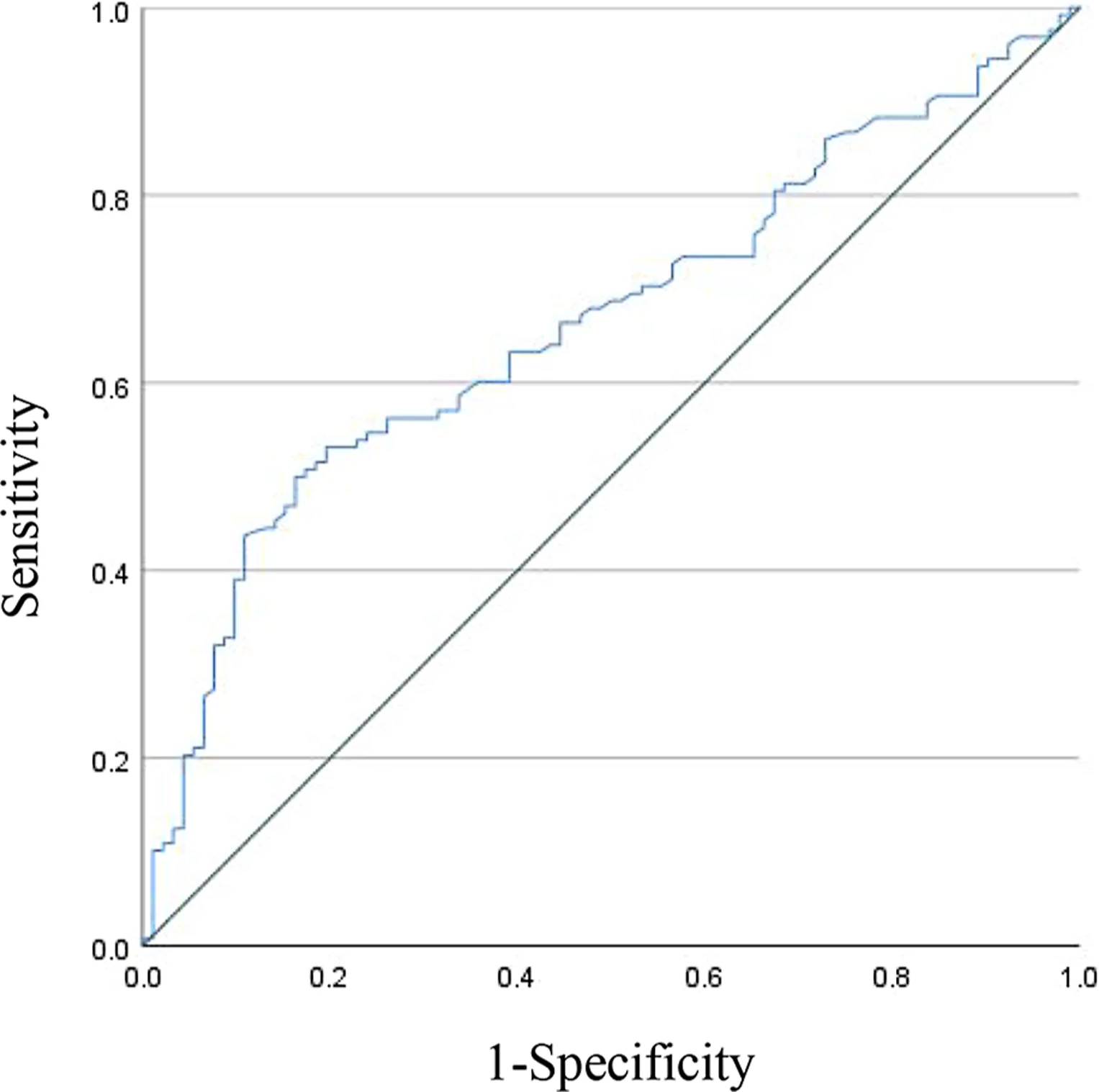
Receiver operating characteristic (ROC) curve of the logistic regression model for predicting falls. The model was constructed based on TUG time, age, and MRC sum score.

### Sensitivity and Subgroup Analyses

When falls were redefined as “≥2 events” (frequent fallers, *n* = 58), the final model retained TUG time and MRC score, but age did not enter the model (*p* = 0.09). Excluding patients using walking aids at baseline (*n* = 31) did not substantially alter the results. Subgroup analyses showed that in limb-onset patients, TUG time and MRC score had stronger predictive effects (OR = 1.095 and 0.971, respectively); in bulbar-onset patients, only TUG time entered the model. Model performance did not differ significantly by Penn UMN score strata.

We further explored the interaction between age and UMN burden by stratifying patients by both age (<57 vs. ≥57 years) and Penn UMN score (<6 vs. ≥6). Among younger patients (<57 years), those with higher UMN burden (Penn UMN ≥6) showed fall rates of 68.25% compared to 32.61% in those with lower UMN burden (*p* = 0.028). While no significant interaction was detected, the limited sample size in stratified subgroups precludes definitive conclusions. This interaction warrants further investigation in larger cohorts.

## Discussion

This prospective cohort study provides a comprehensive characterization of gait abnormalities in ALS and their association with fall risk and functional decline. The main findings are as follows: (1) ALS patients exhibit widespread gait impairments consistent with our prior cross-sectional study; (2) 57.6% of patients fell within 12 months; (3) prolonged TUG time, younger age, and lower MRC score independently predict falls, though model discrimination is modest (AUC = 0.665); and (4) gait parameters, particularly gait speed and TUG time, correlate more strongly with time to wheelchair use than with fall count.

The key incremental contribution of this study lies in the translation of cross-sectional gait descriptors into prospective predictors of clinically meaningful outcomes. While our previous work [10] and others [14, 15] have established that ALS patients exhibit characteristic gait abnormalities, whether these baseline features predict future falls and functional decline has remained uncertain. By systematically following a large cohort over 12 months, we demonstrate that specific gait parameters – particularly TUG time – not only differentiate ALS from healthy controls but also carry prognostic information for fall risk and time to wheelchair use. This shift from cross-sectional characterization to prospective prognostication represents the principal advance of this work. Furthermore, the modest performance of our prediction model provides important benchmark data for future studies and underscores the multifactorial nature of falls, highlighting the need for multimodal risk assessment beyond gait analysis alone.

### Gait Abnormalities in ALS

ALS patients in this cohort exhibited pronounced gait abnormalities across all functional domains, replicating our previous cross-sectional findings [10] and extending the literature [14, 15]. Gait impairment in ALS arises from a combination of UMN and lower motor neuron dysfunction. UMN signs including spasticity, hyperreflexia, and impaired interlimb coordination contribute to reduced gait speed, altered rhythm, and diminished automaticity of locomotor control. Lower motor neuron signs, in contrast, manifest as distal muscle weakness affecting ankle dorsiflexion (foot drop) and plantarflexion (reduced push-off power), as well as proximal weakness compromising hip flexion and stabilization during stance. These dual pathological processes result in a characteristic spatiotemporal phenotype: shortened stride length, reduced cadence, prolonged double support, widened step width, and diminished swing-phase velocity – all of which were observed in our cohort.

The largest effect sizes in our study were observed for cadence, stride speed, and swing speed – parameters reflecting rhythmic control and propulsion – suggesting that these measures may be particularly sensitive to ALS-related gait impairment. The substantial reductions in these parameters (effect sizes |r| ranging from 0.51 to 0.72) indicate that ALS profoundly affects the central generation and execution of rhythmic locomotor patterns, beyond what would be expected from weakness alone.

Comparing our findings with other neurodegenerative conditions provides further context. In Parkinson's disease (PD), gait impairment is characterized by reduced arm swing, shuffling steps, and freezing of gait, primarily driven by basal ganglia dysfunction and dopaminergic deficits [16]. In contrast, ALS gait abnormalities are more prominently related to muscle weakness and spasticity, with less prominent festination or freezing. Unlike PD, where dopaminergic medication can partially restore gait, ALS lacks effective pharmacotherapy for gait symptoms, underscoring the importance of rehabilitation and fall prevention. In contrast to stroke survivors, who often exhibit hemiparetic gait with marked asymmetry, ALS patients typically show more symmetric bilateral involvement – a pattern reflected in our observation that asymmetry indices were actually lower in ALS patients than in healthy controls, likely due to the bilateral and relatively symmetrical nature of motor neuron degeneration in the spinal form of the disease.

The apparent preservation of step height in our cohort – showing no statistically significant difference between ALS patients and healthy controls – may seem counterintuitive given that foot drop and tripping are among the most common early gait complaints in ALS. Several explanations may account for this observation. First, step height is measured during walking, where patients may adopt compensatory strategies (e.g., hip hiking or circumduction) to maintain foot clearance despite ankle dorsiflexor weakness. Second, our cross-sectional measurement captures a single snapshot in time, and patients with more severe foot drop who could no longer compensate may have been excluded if they could not complete the gait test. Third, the absence of a group-level difference does not preclude the clinical importance of foot drop; rather, it suggests that step height as measured in a controlled laboratory setting may not be the most sensitive indicator of this clinical phenomenon. We, therefore, do not interpret these findings as evidence that vertical clearance is clinically unimportant. Instead, we emphasize that both horizontal propulsion (push-off) and vertical clearance (foot clearance) are affected in ALS, and that their relative contributions may vary across disease stages and individuals. The preservation of group-level-step height in our cohort likely reflects effective compensation rather than biological sparing of ankle dorsiflexor function.

### Fall Rate and Predictors

The 12-month fall rate of 57.6% is higher than previously reported rates of 33–45% [4, 17], likely reflecting the longer observation period and inclusion of patients with varying disease stages. TUG time emerged as the strongest predictor, consistent with prior studies demonstrating its utility in fall prediction [18, 19]. Each 1-s increase in TUG time was associated with an 8.3% increase in fall odds. The TUG test integrates sit-to-stand transitions, walking, turning, and balance control – core components affected in ALS – making it a practical clinical screening tool.

The association between lower MRC score and increased fall risk is intuitive as muscle weakness directly compromises stability and compensatory capacity. Interestingly, the effect persisted after adjusting for TUG time, suggesting that the MRC score captures additional aspects of neuromuscular function not fully reflected by performance-based tests.

The inverse relationship between age and fall risk – younger patients being more prone to falls – warrants careful interpretation. While counterintuitive, this finding should be interpreted with caution. We explored whether this association might be explained by more prominent UMN involvement in younger patients; however, stratified analysis did not reveal a significant interaction between age and UMN burden in predicting falls (Sensitivity and Subgroup Analyses). Other possible explanations include more aggressive disease phenotypes, higher levels of physical activity, and greater exposure to fall-prone situations in younger patients, whereas older patients may adopt more cautious gait strategies or use walking aids earlier [20]. This remains a hypothesis-generating observation requiring replication in independent cohorts with objective activity monitoring.

### Model Performance and Interpretability

The modest discrimination (AUC = 0.665) and low specificity (52.2%) indicate that our model, while statistically significant, has limited clinical utility as a standalone fall prediction tool. Several factors may explain the suboptimal performance. First, falls are multifactorial events influenced by environmental factors (flooring, lighting, obstacles), psychological factors (fear of falling, risk-taking behavior), medication use, and caregiver supervision, none of which were captured in our model [21]. Second, we excluded patients unable to walk independently, limiting generalizability to more advanced disease stages. Third, cognitive impairment, which has been shown to increase gait variability and fall risk in ALS, was not systematically assessed. Mild cognitive and behavioral impairment is common in ALS, even among patients classified as cognitively normal according to current diagnostic criteria, and has recently been shown to independently predict disease progression and survival. Executive dysfunction and impaired attention are plausible contributors to gait instability, particularly during complex walking conditions or when negotiating environmental obstacles. Fourth, the relatively modest sample size may have limited model stability.

It is important to emphasize that the current model, given its limited discrimination, is not ready for standalone clinical deployment. Rather, its primary value lies in: (1) confirming that simple clinical measures (TUG, MRC) capture meaningful fall risk information, (2) providing quantitative benchmark data for future model development, and (3) highlighting the substantial gap between single-modality gait assessment and the complex reality of falls in ALS. We view this work as a foundation for subsequent multimodal prediction studies, not as a finished clinical tool.

Despite these limitations, our findings offer several clinical insights. The TUG test, requiring minimal equipment and expertise, may be considered part of routine ALS assessments. A TUG time exceeding 13.5 s (the median in fallers) may warrant fall prevention interventions, as a low-risk, low-cost screening signal. MRC sum score should not be limited to lower limbs as upper limb and trunk strength contribute to overall stability and compensatory strategies. Notably, younger patients – often perceived as lower risk – deserve targeted fall prevention education.

### Gait Parameters and Time to Wheelchair Use

The stronger correlations between gait parameters and time to wheelchair use (compared to fall count) suggest that quantitative gait measures may better capture the progressive erosion of walking capacity that culminates in wheelchair dependence. Gait speed and TUG time, with correlations of 0.366 and −0.381, respectively, emerged as the most robust correlates. These parameters integrate multiple aspects of motor function and may serve as surrogate markers for functional decline in clinical trials. The absence of correlation between gait parameters and ALSFRS-R decline rate may reflect that ALSFRS-R captures broader functional domains (bulbar, respiratory, upper limb) beyond gait.

While time to wheelchair use provides a clinically meaningful indicator of functional decline, it should be interpreted with caution. Wheelchair initiation is influenced by multiple non-motor factors, including patient preference, caregiver support, accessibility of the home environment, and local clinical practices regarding wheelchair prescription. These factors were not systematically captured in our study. Nevertheless, the robust correlations between gait parameters and time to wheelchair use – consistent across multiple parameters and substantially stronger than those with fall count – suggest that this outcome does reflect genuine progressive loss of walking capacity beyond purely social or logistical determinants. We defined time to wheelchair use as the transition to full-time wheelchair dependence, as partial or situational wheelchair use is common in ALS and may be influenced by factors such as distance to destination, fatigue, or social context. We acknowledge that this binary classification does not capture the gradual nature of mobility decline, and that many patients continue to ambulate short distances even after acquiring a wheelchair.

### Comparison with Previous Studies

Montes et al. [18]reported an AUC of approximately 0.68 for TUG in predicting falls in ALS, closely matching our findings. Dubbioso et al. [11] demonstrated that cognitive impairment exacerbates gait variability and fall risk, suggesting that incorporating cognitive assessments might improve our model. Compared to Parkinson’s disease, where gait-based fall prediction models often achieve AUC >0.80 [16], ALS models tend to underperform, likely reflecting the greater clinical heterogeneity and the rapid progression of multiple motor domains simultaneously.

### Limitations

Several limitations should be acknowledged. First, the single-center design and exclusion of non-ambulatory patients limit generalizability to broader ALS populations. Second, the absence of cognitive and behavioral assessments – a critical limitation – likely under-estimated fall risk. Given that mild cognitive and behavioral impairment is common in ALS [22] and has been associated with both gait instability and fall risk, this omission likely contributed to the modest performance of our prediction model and represents a priority for future research. Third, the 12.5% attrition rate may have introduced bias, although sensitivity analyses suggested robustness. Fourth, the modest model performance precludes clinical deployment without further validation. Fifth, we did not capture environmental or psychosocial fall risk factors. Sixth, the matched healthy controls were derived from a historical database, potentially introducing temporal confounding despite propensity score matching. Seventh, we acknowledge that forward stepwise logistic regression may have resulted in unstable variable selection; penalized regression methods with bootstrap validation would provide more robust estimates in future studies. Eighth, our prediction model was developed and internally validated within the same cohort; external validation in an independent cohort is essential before any model can be considered for clinical translation. Ninth, we used LOCF for imputing missing data, which is no longer considered optimal; however, the low attrition rate and the pro-tocol-defined 12-month follow-up window mitigate the risk of substantial bias. Tenth, we did not stratify limb-onset patients by cervical versus lumbar onset. Subgroup analysis comparing cervical versus lumbar onset did not reveal significant differences in gait parameters or fall rates; however, we acknowledge that recall bias – particularly in patients with prominent UMN symptoms who may not recognize subtle early limb stiffness – and the relatively advanced disease stage at presentation may have limited the sensitivity of this analysis. Whether cervical versus lumbar onset influences gait patterns in earlier-stage disease warrants further investigation. Finally, genetic testing was not systematically performed, and NIV use at baseline was not recorded, though patients with clinically significant respiratory insufficiency were excluded from participation.

### The Psychosocial Complexity of Fall Risk in ALS

Beyond clinical and biomechanical predictors, a crucial dimension that our quantitative model cannot capture is the psychosocial and behavioral factors that profoundly influence fall risk in ALS. Many patients struggle to accept their diagnosis and actively resist the progressive loss of independence. This may manifest as reluctance to adopt gait aids, refusal of wheelchair use, or persistence in risky activities to “prove” their continued capability. These behaviors are not simply non-adherence; they represent a deeply human response to a devastating diagnosis. Clinicians working with ALS patients recognize that falls often occur not because patients lack insight, but precisely because they have insight and are fighting to maintain autonomy. Our quantitative model, while capturing biomechanical risk factors, is necessarily blind to this dimension. We, therefore, agree with the clinical perspective that future fall prevention strategies must integrate not only motor assessment but also psychological support, goal-oriented counseling, and shared decision-making around mobility aids. This is not a limitation of our study alone, but a fundamental challenge in ALS care that warrants dedicated research.

### Future Directions

To improve fall prediction, future studies should adopt a multimodal approach integrating quantitative gait analysis with cognitive assessments (ECAS, MoCA), dual-task gait testing [23], and continuous real-world monitoring using wearable sensors [24]. Crucially, future research must also address the psychosocial and behavioral dimensions of fall risk – including acceptance of diagnosis, attitudes toward assistive devices, and the patient's struggle to maintain independence – through qualitative and mixed-methods approaches that complement quantitative modeling. Machine learning methods (random forests, gradient boosting, neural networks) may uncover complex, non-linear relationships among multiple gait parameters that traditional regression models miss [25]. Multi-center external validation in larger cohorts is essential before any model can be translated into clinical practice.

## Conclusion

This prospective study confirms widespread gait abnormalities in ALS and demonstrates that TUG time, younger age, and lower MRC score are associated with falls with modest predictive accuracy. The moderate predictive performance highlights the multifactorial nature of falls and the need for comprehensive, multi-domain risk assessment. Gait speed and TUG time warrant further evaluation as potential surrogate markers for progressive functional decline toward wheelchair dependence. Future research integrating cognitive evaluation, continuous monitoring, and advanced analytical approaches may yield more robust fall prediction tools for personalized ALS care. Future research integrating cognitive evaluation, continuous monitoring, and attention to psychosocial factors, particularly the patient's struggle to maintain independence and acceptance of assistive devices, may yield more robust fall prediction and prevention strategies for personalized ALS care.

## Acknowledgment

We gratefully acknowledge the support of the Clinical Research Unit of the Clinical Efficacy Validation Platform, National Facility for Translational Medicine (PUMCH).

## Statement of Ethics

The study was approved by the Ethics Committee of Peking Union Medical College Hospital (HS-3076). All participants provided written informed consent.

## Conflict of Interest Statement

The authors have no conflicts of interest to declare.

## Funding Sources

This work was funded by the National Science and Technology Major Project (2022ZD0118003) and CAMS Innovation Fund for Medical Sciences (CIFMS 2021-I2M-1-003) and supported by the Ministry of Industry and Information Technology of China.

## Author Contributions

Nan Hu: methodology, investigation, data curation, writing-original draft, formal analysis. Ning Su: investigation, validation, visualization. Dingding Zhang: software, investigation. Jiangxia Zhang: data curation. Kai Wang, Yuming Xu, Zhenzhong Li, Lihua Wang, Bo Wu, Lan Chu, Yinzhou Wang, and Hong Jiang: investigation, data curation. Zhengqi Lu: resources, investigation. Jian Wu: investigation. Xiangmin Fan: investigation. Fei Han: investigation. Jing Yuan: investigation. Yicheng Zhu: writing-review and editing, conceptualization, supervision. Mingsheng Liu: conceptualization, funding acquisition, project administration, writing-review and editing.

## Data Availability

All data generated or analyzed during this study are included in this article and its supplementary material files. Further enquiries can be directed to the corresponding author.
